# ZIF-8 Hydrogel-Mediated Regulation of Macrophage Phenotype Accelerates Frostbite Wound Healing

**DOI:** 10.3390/biomedicines14010051

**Published:** 2025-12-25

**Authors:** Ge Lou, Yutong Li, Jinyu Zhao, Huihui Shao, Xianfu Wu, Heying Jin, Jianpeng Guo, Zhonggao Gao, Xing Jin, Mingji Jin, Shuangqing Wang

**Affiliations:** 1 Key Laboratory of Natural Medicines of the Changbai Mountain, Ministry of Education, College of Pharmacy, Yanbian University, Yanji 133002, China; 2024051191@ybu.edu.cn (G.L.); gjp807@ybu.edu.cn (J.G.); 2 National Institutes for Food and Drug Control, Beijing 102629, China; liyutongvincent@163.com (Y.L.); wuxf99@163.com (X.W.); 3 State Key Laboratory of Bioactive Substance and Function of Natural Medicines, Institute of Materia Medica, Chinese Academy of Medical Sciences and Peking Union Medical College, Beijing 100050, China; zhaojinyu@cmu.edu.cn (J.Z.); shaohuihui@imm.ac.cn (H.S.); zggao@imm.ac.cn (Z.G.); 4 Xiangya Medical College, Central South University, Changsha 410013, China; yizhaohuang02@gmail.com; 5 Department of Ultrasonic Medicine, Yanbian University Hospital, Yanji 133002, China

**Keywords:** MOFs, frostbite, oxyresveratrol, hyaluronic acid, macrophage

## Abstract

**Background**: Frostbite injury creates an ischemic, hypoxic, and acidic microenvironment that often triggers severe oxidative stress and inflammation. Current therapeutic approaches are limited by low drug delivery efficiency and an inability to adequately regulate multiple pathological pathways. Although oxyresveratrol (OR) exhibits excellent antioxidant and anti-inflammatory activities, its application is hampered by poor aqueous solubility and low stability. **Methods**: We constructed Oxyresveratrol@Zeolitic Imidazolate Framework-8 nanoparticles (OR@ZIF-8) and further embedded them in a sodium hyaluronate (HA) matrix to form an OR@ZIF-8@HA composite hydrogel. The physicochemical properties and pH-responsive drug release behavior of the system were characterized. Its antioxidant activity, ability to promote cell migration, and capacity to modulate macrophage polarization were evaluated in cellular assays. The therapeutic efficacy was further investigated using a mouse frostbite model, with wound repair analyzed via histological staining. **Results**: The OR@ZIF-8 nanoparticles achieved a cumulative release rate of 75.46 ± 3.68% under acidic conditions within 36 h. In vitro experiments demonstrated that the formulation significantly scavenged TNF-α and IL-6, by 161.85 ± 19.43% and 125.37 ± 12.65%, respectively, and increased the level of IL-10 by 44.97 ± 4.57%. In a scratch assay, it promoted wound healing, achieving a closure rate of 97.55 ± 2.77% after 36 h. In vivo studies revealed that the OR@ZIF-8@HA treatment group achieved a wound healing rate of 96.14 ± 4.12% on day 14. **Conclusions**: The OR@ZIF-8@HA composite hydrogel effectively overcomes the limitations of OR application via intelligent pH-responsive delivery. Through synergistic multi-mechanistic actions, it significantly accelerates frostbite wound healing, offering a novel and efficient therapeutic strategy for frostbite management.

## 1. Introduction

Frostbite is a common tissue-damaging disease in cold environments, predominantly affecting populations in plateaus, polar regions, and those engaged in outdoor work in winter [1]. Its core pathological features involve hypothermia-induced vasospasm and microcirculatory dysfunction in the skin and subcutaneous tissues, which subsequently trigger ischemia-hypoxia, oxidative stress overload, and an exaggerated inflammatory response [2]. Frostbite can present with skin redness, swelling, and blisters. Severe frostbite is accompanied by tissue necrosis and infection, and may even require amputation to control the condition, seriously affecting patients’ quality of life [3]. Currently, the clinical management of frostbite primarily involves insulation and gradual rewarming, debridement, infection control, and the application of topical growth factors. However, growth factors are characterized by short half-lives, with their local concentrations prone to dilution by the wound microenvironment, thereby limiting their ability to sustainably promote cell proliferation and migration [4]. Traditional anti-inflammatory drugs have poor targeting, are prone to causing systemic side effects, and cannot simultaneously regulate the multi-step pathological damage during frostbite repair [5,6]. Currently, there is no effective treatment for frostbite. Therefore, developing a novel multi-targeted repair preparation that can precisely regulate the wound microenvironment and simultaneously modulate oxidative stress, the inflammatory response, and tissue regeneration has become a key priority in the field of frostbite treatment.

Frostbite wound healing is a complex dynamic process involving the regulation of oxidative stress, resolution of inflammation, cell migration and proliferation, and collagen remodeling. The dysregulation of multiple core links in this process is a key factor contributing to delayed repair [7]. Following hypothermic injury, vascular endothelial cells are damaged, leading to massive accumulation of local reactive oxygen species (ROS). ROS not only directly cause oxidative damage to cells but also activate inflammatory signaling pathways, exacerbating the inflammatory cascade [8]. Additionally, wound macrophages undergo excessive polarization toward the pro-inflammatory M1 phenotype, secreting large amounts of pro-inflammatory cytokines such as tumor necrosis factor-α (TNF-α) and interleukin-6 (IL-6). These cytokines inhibit the migration and proliferation of epidermal cells and impede wound re-epithelialization [9]. Given the aforementioned mechanisms, single-target therapeutic strategies are insufficient for efficient repair, necessitating the development of multifunctional preparations capable of synergistically regulating multiple pathological processes.

In recent years, natural products with prominent pharmacological activities have garnered widespread attention [10]. Shen et al. treated frostbite wounds with triterpenoids isolated from Ganoderma lucidum [11]. However, the low yield and high cost of Ganoderma lucidum limit its further application. OR, a natural polyphenolic compound, exhibits excellent antioxidant and anti-inflammatory activities [12]. It can protect cells from oxidative damage by scavenging ROS [13,14]. Nevertheless, the clinical application of OR is limited by its poor water solubility, susceptibility to oxidative degradation, and low bioavailability; thus, there is an urgent need to improve its pharmacokinetic properties through a carrier system. ZIF-8 has emerged as an ideal carrier due to its unique structural advantages. ZIF-8 possesses a high specific surface area and porous structure, enabling efficient encapsulation of hydrophobic drugs and improved water solubility [15]. Meanwhile, ZIF-8 exhibits pH responsiveness, maintaining structural stability under normal physiological pH [16], but rapidly disassembling to release drugs in the acidic microenvironment of frostbite wounds, thereby achieving on-demand drug release. It is worth noting that the zinc ion component of ZIF-8 has excellent biocompatibility, with degradation products showing low cytotoxicity to cells, making ZIF-8 a suitable molecule for tissue repair [17,18].

Considering the problems associated with nanoparticles, e.g., they are prone to being lost through wound exudate and fail to maintain a moist wound microenvironment, this study further introduced HA hydrogel as the matrix carrier. As a natural polymer polysaccharide, HA exhibits excellent biocompatibility, moisture retention capacity, and wound adhesion [19,20,21]. HA forms a protective film on the wound surface, reducing moisture evaporation and maintaining a moist wound microenvironment. Furthermore, HA forms a stable composite system (OR@ZIF-8@HA) with OR@ZIF-8 nanoparticles. Through the adhesive properties of the hydrogel, nanoparticles are retained at the wound site for extended periods, reducing drug loss and prolonging therapeutic activity.

Building on this, this study innovatively developed an OR@ZIF-8@HA hydrogel composite formulation. The core objective is to simultaneously modulate key pathological processes in frostbite repair, leveraging the synergy between ZIF-8-mediated drug encapsulation and HA-based matrix immobilization. First, OR@ZIF-8 nanoparticles were fabricated using the one-pot method, followed by systematic characterization of their particle size, morphology, encapsulation efficiency (EE), and pH-responsive drug release behavior. Using the mice frostbite model, the in vivo therapeutic efficacy of OR@ZIF-8@HA for frostbite repair was evaluated. Leveraging the synergistic interaction between the dual carriers ZIF-8 and HA as well as the drug OR, this study provides a novel multifunctional nanoformulation for frostbite repair, while also offering new insights into multi-targeted regulation of wound healing.

## 2. Materials and Methods

### 2.1. Materials

Oxyresveratrol (CPC960), 2,2-Diphenyl-1-picrylhydrazyl (DPPH, D807297), 2,2′-Azino-bis(3-ethylbenzothiazoline-6-sulfonic acid) diammonium salt (ABTS^+^, A800764) and Crystal Violet Staining Solution (C0121), Anti-CD86 antibody (ab239075), Anti-mannose receptor antibody (CD206; ab64693), VEGF antibody (ab32152) were purchased from Abcam (Shanghai, China). Zinc nitrate hexahydrate was obtained from Lanzhou Yellow River Institute of Zinc and Magnesium Nanomaterials (Lanzhou, Gansu, China). Hydrogen peroxide (11641-2) was purchased from Beijing TongGuang Fine Chemicals Company (Beijing, China). Ethylene glycol was purchased from Beijing TongGuang Fine Chemicals Company (Beijing, China; no product number provided). Sodium hyaluronate (150–390 kDa; TC23028) was sourced from Kewpie Corporation (Tokyo, Japan). The CCK-8 assay kit (C0038) was purchased from Solarbio (Beijing, China). Calcein AM Cell Viability Assay Kit (C2013M) was purchased from Shanghai Beyotime Biotech. Inc. (Shanghai, China). Immunostaining Permeabilization Buffer with Triton X-100 (P0096), 4% Paraformaldehyde Fix Solution (P0099), and DAPI solution (ready-to-use, C0065) were purchased from Shanghai Beyotime Biotech. Inc. (Shanghai, China) and Beijing Solarbio Science & Technology Co., Ltd. (Beijing, China), respectively. 3M Tegaderm^TM^ Film (3582) was purchased from 3M (St. Paul, MN, USA). All cells were cultured in Dulbecco’s modified Eagle’s medium (DMEM) supplemented with 10% fetal bovine serum (FBS) and 1% penicillin–streptomycin. Cells were maintained in a humidified incubator at 37 °C in an atmosphere containing 5% CO_2_.

The mouse mononuclear macrophage leukemia cell line (RAW264.7) and human immortal keratinocyte line (HaCaT) cells were obtained from the Cell Resource Center, IBMS, CAMS/PUMC.

### 2.2. Preparation of OR@ZIF-8 Nanoparticles

OR@ZIF-8 nanoparticles were synthesized via a one-pot method with minor modifications from previous reports [16]. Briefly, OR was dissolved in DMSO to obtain a stock solution (2.5 mg/mL). Zn(NO_3_)_2_·6H_2_O (40 mg) was dissolved in DMSO (2 mL), followed by addition of 400 μL OR stock solution (containing 1.0 mg OR) and stirring for 15 min at 25 °C. Subsequently, 2-methylimidazole (400 mg) in methanol (2 mL) was added dropwise over 10 min under stirring (1500 rpm), and the mixture was further stirred for 30 min. The precipitate was collected via centrifugation (14,000 rpm, 15 min), washed three times with methanol (5 mL each), and vacuum-dried for 36 h to obtain OR@ZIF-8. Blank ZIF-8 was prepared identically, except that 400 μL of DMSO was added instead of the OR stock solution.

### 2.3. Characterization of OR@ZIF-8 Nanoparticles

#### 2.3.1. Characterization of Particle Size, Polydispersity Index, and Zeta Potential

OR@ZIF-8 nanoparticles were dispersed in ultrapure water. After ultrasonic dispersion, the particle size distribution, polydispersity index (PDI), and zeta potential were determined using a dynamic light scattering (DLS) instrument at 25 °C. Each sample was measured in triplicate, and the average value was calculated.

#### 2.3.2. Transmission Electron Microscopy Characterization

A small amount of OR@ZIF-8 nanoparticle dispersion was dropped onto a carbon-coated copper grid. After air-drying, the morphology and size of the nanoparticles were observed using a transmission electron microscope (TEM) at an accelerating voltage of 100 kV.

#### 2.3.3. Stability Evaluation

Dried OR@ZIF-8 nanoparticles were stored at 4 °C. An appropriate amount of nanoparticles were taken out daily, dispersed in ultrapure water, and ultrasonicated for 5 min to ensure uniform dispersion. Subsequently, the particle size and PDI of the dispersion were determined using a DLS instrument. Each time point was measured in triplicate, with continuous monitoring for 7 d. The changes in colloidal stability of the nanoparticles during storage at 4 °C were analyzed.

#### 2.3.4. Determination of Drug Loading and Encapsulation Efficiency

The drug loading (DL) and encapsulation efficiency (EE) were calculated as previously described [22,23], OR@ZIF-8 nanoparticles were accurately weighed, dissolved in methanol, and the ZIF-8 framework was ultrasonically disrupted to release OR. The content of OR was determined by high-performance liquid chromatography (HPLC, 1260 Agilent, Waldbronn, Germany).

#### 2.3.5. Drug Release Behavior

The in vitro release behavior of OR@ZIF-8 was evaluated using the dialysis bag method. OR@ZIF-8 nanoparticles were suspended in PBS solutions with different pH values (5.5, 6.5, and 7.4) and loaded into dialysis bags with a molecular weight cutoff of 3500 Da. The dialysis bags were then placed to beakers containing fresh release medium and incubated in a shaker at 37 °C and 100 rpm. Samples were collected at 0.5, 1, 2, 4, 8, 12, 24, and 36 h, with simultaneous supplementation of an equal volume of fresh medium. The concentration of OR in the release medium was determined via HPLC, and the cumulative release rate was calculated.

### 2.4. Determination of In Vitro Antioxidant Activity

#### 2.4.1. Determination of DPPH· Free Radical Scavenging Capacity

DPPH was dissolved in absolute ethanol to prepare a 0.2 mM DPPH ethanol solution. Series solutions of OR and OR@ZIF-8 with concentrations of 5, 10, 20, 30, 40, 50, and 100 μg/mL were prepared using ultrapure water, respectively. 2 mL of the sample solution was mixed with 2 mL of the DPPH ethanol solution, and incubated in the dark at 25 °C for 30 min. The blank group was prepared by replacing the sample solution with absolute ethanol, and the control group was prepared by replacing the DPPH solution with ultrapure water. The absorbance was measured at 517 nm using a UV-Vis spectrophotometer (1800–Vis, Shimadzu, Kyoto, Japan), and the scavenging rate was calculated.

#### 2.4.2. Determination of ABTS^+^ Free Radical Scavenging Capacity

A 7 mM ABTS solution was mixed with an equal volume of 2.45 mM potassium persulfate solution, and incubated in the dark at room temperature for 12 h to form a stock solution. Before use, the stock solution was diluted with absolute ethanol to adjust the absorbance at 734 nm to 0.70 ± 0.02, obtaining the ABTS^+^ working solution. 1 mL of OR or OR@ZIF-8 solution with series concentrations was mixed with 3 mL of the ABTS^+^ working solution, and incubated in the dark at 25 °C for 6 min. The blank group was prepared by replacing the sample solution with ultrapure water. The absorbance was measured at 734 nm, and the scavenging rate was calculated.

#### 2.4.3. Determination of ·OH Free Radical Scavenging Capacity

Solution preparation: 0.75 mM 1,10-phenanthroline solution, 0.75 mM FeSO_4_ solution, and 0.01% H_2_O_2_ solution were prepared. 1 mL of 1,10-phenanthroline solution, 1 mL of FeSO_4_ solution, 1 mL of sample solution, and 1 mL of PBS were added sequentially. After mixing, 1 mL of H_2_O_2_ solution was added, and the mixture was incubated in a 37 °C water bath for 60 min. The control group was prepared by replacing the H_2_O_2_ solution with ultrapure water, and the damage group was prepared by replacing the sample solution with ultrapure water. The absorbance was measured at 536 nm, and the scavenging rate was calculated. Three parallel samples were set for each concentration, and the experiment was repeated three times.

### 2.5. Determination of Cytocompatibility and Protective Effect Against Oxidative Damage

#### 2.5.1. Cell Culture

Human immortalized keratinocytes (HaCaT) and Mouse Monocytic Macrophage Leukemia Cell Line (RAW264.7) were obtained from the Cell Resource Center, IBMS, CAMS/PUMC. HaCaT and RAW 264.7 cells were selected and cultured in DMEM containing 10% FBS and 1% penicillin–streptomycin, in a constant temperature incubator at 37 °C with 5% CO_2_. When cell confluency reached 80%~90%, the cells were digested and passaged using 0.25% trypsin-EDTA solution. Cells in the logarithmic growth phase were selected for subsequent experiments to ensure stable cell activity.

#### 2.5.2. Cell Culture Screening of OR@ZIF-8 Concentration

HaCaT cells in the logarithmic growth phase were seeded into 96-well plates at a density of 1 × 10^4^ cells/well, with 100 μL of medium added to each well. The cells were cultured at 37 °C for 24 h until fully adherent. The original medium in each well was discarded, and fresh medium containing OR@ZIF-8 at series concentrations was added separately, with 6 replicate wells set for each group. A blank medium group was set simultaneously for background correction. After 24 and 48 h of culture, 10 μL of CCK-8 reagent was added to each well, followed by further incubation for 2 h. The absorbance of each well was measured at 450 nm using a microplate reader (Biotek, Winooski, VT, USA), and cell viability was calculated.

#### 2.5.3. Protective Effect of OR@ZIF-8 Against H_2_O_2_-Induced Oxidative Damage in HaCaT Cells

HaCaT cells in the logarithmic growth phase were seeded into 96-well plates at a density of 1 × 10^4^ cells/well, with 100 μL of DMEM containing 10% FBS added to each well. The cells were cultured at 37 °C with 5% CO_2_ for 24 h until fully adherent. The original medium was discarded, and medium containing 200 μM H_2_O_2_ was added, followed by incubation at 37 °C for 24 h. The experiment was divided into 5 groups: Control group (no H_2_O_2_-induced damage, no drug), Model group, ZIF-8 group, OR group, and OR@ZIF-8 group. Subsequent incubation was performed for 24 and 48 h, respectively. The medium was discarded, 10 μL of CCK-8 reagent was added, and the cells were incubated at 37 °C for 2 h. The absorbance of each well at 450 nm was measured using a microplate reader, and cell viability was calculated.

#### 2.5.4. Verification by Live Cell Fluorescence Imaging

HaCaT cells were seeded into 6-well plates at a density of 5 × 10^4^ cells/well, with 1 mL of medium added, and cultured at 37 °C for 24 h until adherent. The grouping and treatment were performed as described in Section 2.5.3, with incubation times adjusted to 24, 48, and 72 h. The medium in each well was discarded, and the cells were washed 3 times with PBS preheated to 37 °C. PBS containing 5 μM Calcein-AM was added, and the cells were incubated in the dark at 37 °C for 20 min. Unbound probes were removed by washing 3 times with PBS. A confocal laser scanning microscope (CLSM, Biotek, Winooski, VT, USA, excitation wavelength: 494 nm; emission wavelength: 517 nm) was used to observe and capture images, to intuitively verify the promoting effect of OR@ZIF-8 on the survival and proliferation of HaCaT cells with oxidative damage.

### 2.6. Determination of HaCaT Cell Migration Ability

#### 2.6.1. Cell Scratch Assay

HaCaT cells in the logarithmic growth phase were seeded into 6-well plates at a density of 2 × 10^5^ cells/well, with 1 mL of DMEM containing 10% FBS added. The cells were cultured at 37 °C with 5% CO_2_ until the cell confluency exceeded 90%. Three parallel wounds were scratched vertically along the bottom of each well using a sterile 200 μL pipette tip. The wells were gently washed twice with PBS to remove detached cells. Each group was refreshed with medium containing the corresponding drug and incubated at 37 °C. Photographs were taken using an inverted microscope at 0, 12, and 36 h of incubation, respectively. The scratch width at each time point was measured using Image J software (Version 1.53t, National Institutes of Health), and the healing rate was calculated.

#### 2.6.2. Transwell Migration Assay

Transwell chambers (costar 3422, 8.0 µm pore size) were selected, and 200 μL of DMEM was added to the lower chamber for equilibration for 30 min. HaCaT cells were starved for 12 h using serum-free DMEM, and the cell concentration was adjusted to 5 × 10^4^ cells/mL. According to the above 5 groups, 200 μL of the cell suspension was added to the upper chamber respectively. The chambers were placed into 24-well plates and incubated at 37 °C with 5% CO_2_ for 12 h. After incubation, the chambers were taken out, and the non-migrated cells in the upper chamber were gently wiped off with cotton swabs. The migrated cells were fixed with 4% paraformaldehyde for 30 min and stained with 0.1% crystal violet solution for 20 min, followed by washing with PBS until the background was colorless. Five fields of view were randomly selected under an inverted microscope, and the number of cells migrated to the lower surface of the chamber in each field was counted. The average value was taken as the number of migrated cells in each group. Three replicate wells were set for each group, and the experiment was repeated three times as independent experiments.

### 2.7. Detection of Inflammatory Cytokines

RAW264.7 cells were seeded into 96-well plates at a density of 1.5 × 10^5^ cells/well, with 100 μL of medium added to each well. The cells were cultured at 37 °C for 24 h until adherent. The original medium was discarded, and medium containing 1 μg/mL lipopolysaccharide (LPS) was added, followed by incubation at 37 °C for 24 h. According to the aforementioned grouping, the corresponding samples were added to each well. After 24 h of incubation, cell supernatants from each group were collected and centrifuged at 1200 rpm for 10 min to remove residual cell debris. The supernatants were stored at −80 °C. Experiments were performed strictly following the instructions of the ELISA kits for TNF-α, IL-6, and interleukin-10 (IL-10). Six replicate wells were set for each group.

### 2.8. Macrophage Polarization

RAW264.7 cells were seeded into culture dishes at a density of 5 × 10^4^ cells/well and cultured for 24 h until adherent. The original medium was discarded, and medium containing 1 μg/mL LPS was added, followed by incubation at 37 °C for 24 h. According to the aforementioned grouping, the corresponding samples were added to each well. After 24 h of incubation, the cells were washed with PBS, fixed with 4% paraformaldehyde for 30 min, and permeabilized with 0.1% Triton X-100 for 20 min. Primary antibodies against Cluster of Differentiation 86 (CD86) or Cluster of Differentiation 206 (CD206) (diluted at 1:200) were added, and the cells were incubated overnight at 4 °C. After washing with PBS, the corresponding fluorescent secondary antibodies were added, followed by incubation at room temperature in the dark for 1 h. Cell nuclei were stained with 5 μg/mL DAPI for 5 min. Images were captured using a CLSM, and the fluorescence intensities of CD86 and CD206 were quantified using Image J software.

### 2.9. Preparation of OR@ZIF-8@HA Hydrogel

To prepare the OR@ZIF-8–loaded HA hydrogel (OR@ZIF-8@HA), 1 mg OR@ZIF-8 was first dispersed in 1 mL pH 7.4 PBS by vortexing for 1 min, followed by bath sonication for 5 min to obtain a homogeneous suspension. Subsequently, 20 mg HA was gradually added to the above suspension under gentle stirring to avoid aggregation, yielding a final 2% HA (*w*/*v*). The mixture was then allowed to hydrate at room temperature for 6 h (with brief manual mixing every 1 h until a uniform gel-like formulation was obtained. No chemical crosslinker was used. The gel-like network was formed via hydration-induced physical gelation and polymer chain entanglement of HA at 2% (*w*/*v*) in PBS. The OR@ZIF-8@HA hydrogel was freshly prepared prior to experiments and used on the same day. For controls, blank HA hydrogel was prepared using the same procedure without adding nanoparticles.

### 2.10. Characterization of OR@ZIF-8@HA Hydrogel

#### 2.10.1. Swelling Behavior

Hydrogel samples were freeze-dried to constant mass to obtain the initial dry weight. Each dried sample was immersed in pH 7.4 PBS at 37 °C under static conditions (no shaking) without PBS replacement. At predetermined time points, samples were removed, gently blotted with filter paper to remove surface liquid, and weighed to obtain their wet weight. From these values, the degree of swelling was calculated.

#### 2.10.2. Mass Loss in PBS

For mass loss evaluation, freeze-dried samples with known initial dry weight were incubated in pH 7.4 PBS at 37 °C under static conditions. At scheduled time points, samples were collected (n = 3), freeze-dried again to constant mass, and the mass remaining was calculated. All measurements were performed in triplicate.

### 2.11. Study on Mouse Frostbite Model

SPF-grade BALB/c mice (male, 6–8 weeks old, weight 20–22 g) were selected and housed in a barrier environment with a temperature of (22 ± 2) °C and humidity of (50 ± 5)%, the mice had free access to food and water. All animal experiments were approved by the Laboratory Animal Ethics Committee in the Institute of Materia Medica and Peking Union Medical College (IMM-S-25-0593). Experiments were performed strictly adhering to the 3R principles (Replacement, Reduction, Refinement). The mice were fasted for 6 h before the experiment and anesthetized via inhalation of 5% isoflurane for 5 min [24,25,26]. After the disappearance of the corneal reflex, the hair on the right hind limb of the mice was removed (hair removal area with a diameter of approximately 1.5 cm) and the skin was cleaned with normal saline. An iron rod (diameter 0.8 cm), pre-chilled in liquid nitrogen was applied to the mouse back for 50 s. A liquid-nitrogen-induced frostbite wound model was established after 24 h. Mice with established wounds were randomly divided into 5 groups: Model group, Positive group (3M dressing), HA group, OR@ZIF-8 group, and OR@ZIF-8@HA group. Each group contained 6 mice. Drug administration was performed once daily. On day 14, the mice were euthanized. Skin tissues from the wound area were collected for histopathological examination, including Hematoxylin and Eosin (H&E) staining, Masson staining, and macrophage phenotype detection.

### 2.12. Statistical Analysis

All experimental data in this study were expressed as “mean ± standard deviation (SD)”. Data statistics and graphing were performed using GraphPad Prism 9.0 software. For comparisons of multiple groups at a single time point, one-way analysis of variance (One-way ANOVA) was used, and pairwise comparisons between groups were performed using *t*-tests. All experiments were independently repeated at least three times.

## 3. Results

### 3.1. Physicochemical Characterization of Nanoparticles

**Figure 1:**
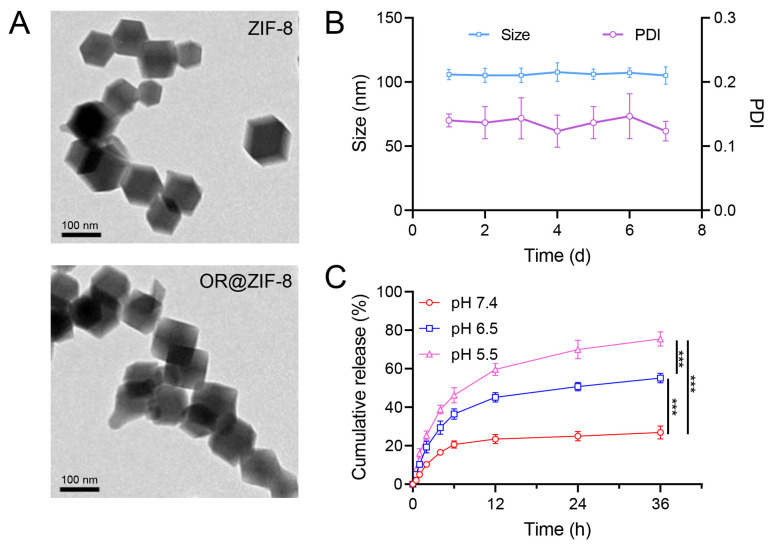
Characterization and sustained release of drugs from OR@ZIF-8 nanoparticles. (**A**) TEM image of ZIF-8 and OR@ZIF-8, scale = 100 nm. (**B**) Size and PDI of OR@ZIF-8. (**C**) Cumulative release rate of OR@ZIF-8 at pH 7.4, 6.5, and 5.5 (n = 3, means ± SD), *** *p* < 0.001.

To confirm the successful construction of the nanocarrier and verify that its physicochemical parameters were suitable for biomedical applications, we conducted a series of experiments. The physicochemical characterization results of OR@ZIF-8 nanoparticles are shown in Figure 1. The average particle size of the ZIF-8 nanoparticles was 91.3 ± 4.1 nm, with a narrow unimodal distribution. After OR loading, the average particle size of OR@ZIF-8 increased to 106.1 ± 5.6 nm, monodispersity was maintained. This indicated successful OR loading without the induction of nanoparticle aggregation, meaning the particle size was suitable for particle size basis for its penetration and distribution in skin wounds. The zeta potential of ZIF-8 was 22.8 ± 3.7 mV; this value decreased to 15.6 ± 2.4 mV for OR@ZIF-8. However, this value remains within a high positive range, suggesting that the nanoparticles retained good dispersion stability after OR loading. This could prevent abnormal drug release or sudden particle size changes caused by aggregation during storage. In the TEM images (Figure 1A), both ZIF-8 and OR@ZIF-8 exhibit regular polyhedral (rhombic dodecahedral) structures with uniform particle morphology. Comparative observation revealed that the particle outline of OR@ZIF-8 was similar to that of ZIF-8, visually confirming the successful loading of OR in ZIF-8 without destroying the crystalline morphology of ZIF-8. During the 7 d monitoring period, the OR@ZIF-8 particle size remained at approximately 100 nm, and the PDI remained within the range of 0.1–0.2 without significant fluctuations (Figure 1B). OR@ZIF-8 exhibited excellent colloidal stability, meeting the requirements for the dispersion and uniformity of the formulation in long-term storage and clinical applications.

### 3.2. Drug Loading and Encapsulation Efficiency

We quantified the drug loading capacity and preparation efficiency of the nanocarrier to ensure the feasibility of the loading process. DL and EE are key indicators for evaluating the drug-loading capacity of nanocarriers, directly reflecting the EE of ZIF-8 for OR and the drug bioavailability of the formulation. The DL of OR@ZIF-8 was approximately 5.47 ± 0.75%, and the EE reached approximately 89.07 ± 3.37%. This indicated that ZIF-8 exhibited high-efficiency loading capacity for OR, which could provide a sufficient OR dose for subsequent drug delivery to frostbite wounds, reducing drug waste in topical formulations and the need for frequent administration.

### 3.3. pH-Responsive In Vitro Drug Release Behavior

To verify that the carrier can achieve “on-demand” intelligent drug release in response to the acidic microenvironment of frostbite wounds, we conducted a series of experiments. As shown in Figure 1C, in the simulated normal skin environment at pH 7.4, the cumulative release rate of OR was only 26.87 ± 1.36% over 36 h. In contrast, in acidic environments (pH 6.5 and pH 5.5), the cumulative release rate of OR increased significantly, reaching 55.13 ± 2.45% and 75.46 ± 3.68% respectively, at 36 h. This result indicated that OR@ZIF-8 exhibited a distinct pH-responsive release profile: it could increase OR release in the acidic microenvironments of frostbite wounds while maintaining low drug release in normal skin environments. This not only ensures effective local drug concentration at the wound site but also minimizes off-target adverse effects effects on normal skin.

### 3.4. In Vitro Antioxidant Activity

To validate the core therapeutic mechanism, we directly assessed the free radical scavenging capability of both the drug and the drug-loaded system, targeting the key pathological role of oxidative stress in frostbite repair. To do this, it was necessary to verify the scavenging capacity of OR@ZIF-8 against different types of free radicals to clarify the retention of OR’s antioxidant activity after encapsulation.

**Figure 2:**
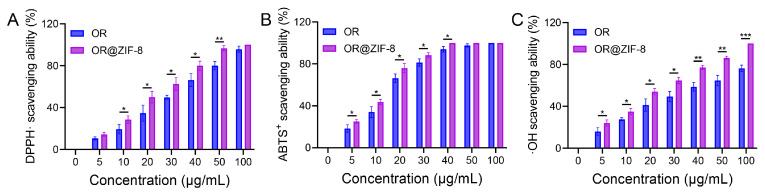
In vitro antioxidant activity of OR@ZIF-8 nanoparticles. (**A**) DPPH· scavenging capacity of OR and OR@ZIF-8. (**B**) ABTS^+^ scavenging capacity of OR and OR@ZIF-8. (**C**) ·OH scavenging capacity of OR and OR@ZIF-8 (n = 3, means ± SD), * *p* < 0.05, ** *p* < 0.01, and *** *p* < 0.001.

As shown in Figure 2A, as their concentration increased (5–100 μg/mL), the scavenging capacity of both OR and OR@ZIF-8 against DPPH· also increased. At multiple concentration points, the DPPH· scavenging capacity of OR@ZIF-8 was significantly higher than that of OR. This indicated that after encapsulation in ZIF-8, the antioxidant activity of OR against lipophilic free radicals was retained or even enhanced. The ABTS^+^ scavenging results are shown in Figure 2B. The ABTS^+^ scavenging capacity of both OR and OR@ZIF-8 increased with concentration. At concentrations of 5, 10, 20, and 30 μg/mL, the ABTS^+^ scavenging capacity of OR@ZIF-8 was significantly better than that of OR, verifying that the antioxidant activity of OR against hydrophilic free radicals was maintained after encapsulation. The ·OH scavenging results are shown in Figure 2C. ·OH is a highly reactive free radical in organisms and is associated with significant cytotoxicity. Furthermore, the scavenging capacity of antioxidants for ·OH is directly related to their potential to alleviate oxidative-stress-induced damage. The ·OH scavenging capacity of both OR and OR@ZIF-8 gradually increased with concentration. At multiple concentration points, the ·OH scavenging capacity of OR@ZIF-8 was significantly higher than that of OR. These results show that OR’s ability to scavenge highly reactive hydroxyl radicals was retained upon ZIF-8 encapsulation. Meanwhile, OR@ZIF-8 enhanced antioxidant efficiency through the synergistic effect between the carrier and the drug, laying a foundation for alleviating oxidative-stress-induced damage in frostbite.

### 3.5. Cytocompatibility and Protective Effect Against Oxidative Damage

To ensure the biological safety of the formulation and confirm its direct protective effects against oxidative stress at the cellular level, we conducted a series of experiments. Cytocompatibility is a core prerequisite for the application of topical formulations to skin wounds. Additionally, subsequent to frostbite, skin tissue is prone to damage due to excessive accumulation of oxidative-stress-induced H_2_O_2_.

**Figure 3:**
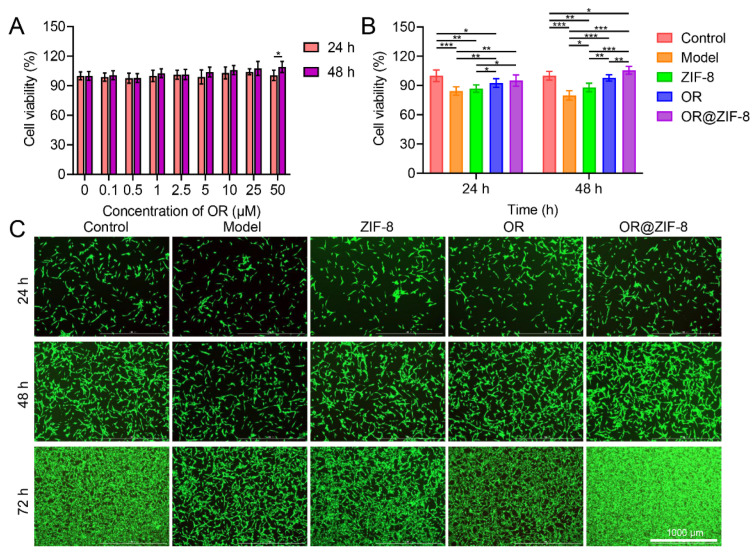
Cytocompatibility and protective effect against oxidative damage. (**A**) Cell viability of OR@ZIF-8 with different OR concentrations ranging from 0 to 50 μM after 24 and 48 h. (**B**) Cell viability at 24 and 48 h (n = 3, means ± SD), * *p* < 0.05, ** *p* < 0.01, and *** *p* < 0.001. (**C**) Live/Dead Cell staining at 24, 48, and 72 h, scale = 1000 μm.

Cell viability was measured after treatment with OR@ZIF-8 containing different concentrations of OR (0–50 μM) for 24 and 48 h to determine the safe concentration range. As shown in Figure 3A, cell viability remained above 85% within the experimental concentration range, indicating that OR@ZIF-8 exhibited no significant cytotoxicity at these concentrations and providing a basis for selecting drug concentrations in subsequent cell experiments. To further evaluate the biocompatibility of OR@ZIF-8, comparisons were made between groups. Compared with the Control group, cell viability in the Model group decreased significantly (Figure 3B). After treatment with ZIF-8, OR, or OR@ZIF-8, cell viability increased to varying degrees. Of these, the cell viability in the OR@ZIF-8 group at 24 and 48 h was significantly higher than that in the Model or OR groups. This suggests that OR@ZIF-8 exerted a superior protective effect on cells damaged by H_2_O_2_-induced oxidative stress, which may be related to the ZIF-8 carrier enhancing the cellular uptake or action efficiency of OR.

Intuitive observation of cell morphology and survival was conducted via live/dead cell staining. In the Control group, cells exhibited intact morphology, uniform fluorescence, and gradually increasing confluency over time (Figure 3C). In the Model group, cell fluorescence was significantly reduced and sparsely distributed. After treatment with ZIF-8 or OR, cell fluorescence was partially recovered. However, at 24, 48, and 72 h, green fluorescence intensity and cell density were significantly higher in the OR@ZIF-8 group than in the other treatment groups. At 72 h, uniform fluorescence was observed across almost the entire field of view. These results further confirm that OR@ZIF-8 could effectively promote the survival and proliferation of H_2_O_2_-damaged cells, laying a foundation for the cellular-level repair of frostbitten skin.

### 3.6. Effect on Cell Migratory Capacity

**Figure 4:**
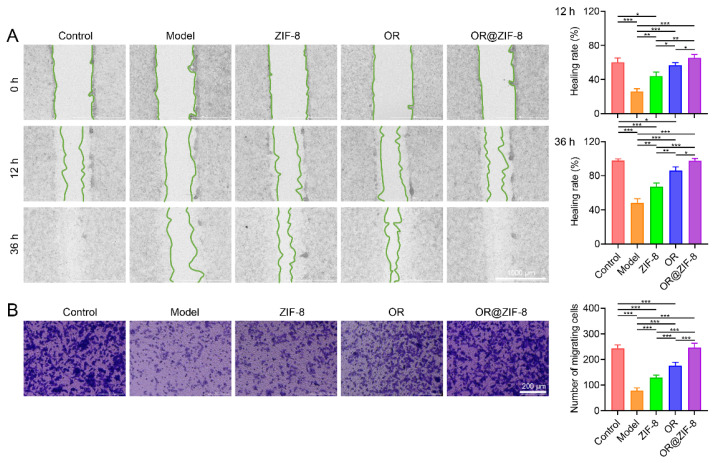
Cell migration ability. (**A**) Wound healing assay at 0, 12, and 36 h (scale = 1000 μm, n = 3, means ± SD), * *p* < 0.05, ** *p* < 0.01, and *** *p* < 0.001. (**B**) Transwell migration assay (scale = 200 μm, n = 5, means ± SD), *** *p* < 0.001.

We evaluated the ability of the formulation to promote cell migration and accelerate wound re-epithelialization. Cell migration is a core process in wound repair. The regulatory effect of OR@ZIF-8 on cell migration was evaluated via the wound healing and Transwell migration assays. As shown in Figure 4A, the scratch width was consistent across all groups at 0 h. After 12 and 36 h, the scratch healing rate of the Model group was significantly lower than that of the Control group, suggesting that injury inhibited cell migration. After treatment with ZIF-8, OR, or OR@ZIF-8, the scratch healing rate increased significantly. Specifically, the healing rate of the OR@ZIF-8 group was 65.38 ± 3.92% at 12 h and 97.55 ± 2.77% at 36 h, which was significantly higher than that of the ZIF-8 and OR groups. This indicated that OR@ZIF-8 could more efficiently promote cell migration to the scratch area. Crystal violet staining showed (Figure 4B) that significantly fewer cells migrated to the lower chamber in the Model group than in the Control group. After treatment with ZIF-8, OR, or OR@ZIF-8, the number of migrated cells increased; among these, the OR@ZIF-8 group had a significantly higher number of migrated cells (246.8 ± 16.4%) compared to the ZIF-8 (129.2 ± 9.6%) and OR groups (176.0 ± 13.1%). These results quantitatively confirmed the enhancing effect of OR@ZIF-8 on cell migratory capacity, which could be important in frostbite wound repair.

### 3.7. Regulatory Effect on Inflammatory Cytokine Secretion

**Figure 5:**
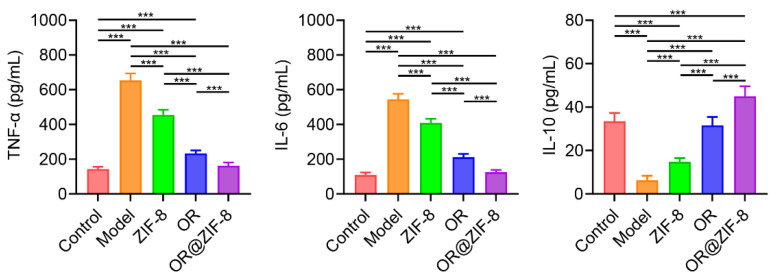
In vitro levels of TNF-α, IL-6, and IL-10 (n = 6, means ± SD), *** *p* < 0.001.

We investigated whether the formulation could inhibit excessive inflammatory responses, thereby creating a favorable environment for tissue repair. During tissue repair, excessive inflammatory responses exacerbate tissue damage, while anti-inflammatory cytokines alleviate inflammation and promote repair. Therefore, the secretion of inflammatory cytokines was assessed to clarify the anti-inflammatory mechanism of OR@ZIF-8. Compared with the Control group, the levels of pro-inflammatory cytokines, such as TNF-α and IL-6, in the Model group were significantly increased, suggesting that injury induced a strong pro-inflammatory response (Figure 5). After treatment with ZIF-8, OR, or OR@ZIF-8, the levels of TNF-α and IL-6 were inhibited to varying degrees. Specifically, the levels of TNF-α and IL-6 in the OR@ZIF-8 group were significantly lower than those in the ZIF-8 and OR groups. IL-10 (an anti-inflammatory cytokine) levels in the Model group were significantly decreased, whereas OR@ZIF-8 treatment significantly increased IL-10 levels compared to ZIF-8 or OR treatment. These results indicated that OR@ZIF-8 could regulate the inflammatory microenvironment through two different pathways—suppressing pro-inflammatory cytokine release and promoting anti-inflammatory cytokine secretion—thus creating favorable conditions for frostbite repair.

### 3.8. Regulatory Effect on Macrophage Polarization

**Figure 6:**
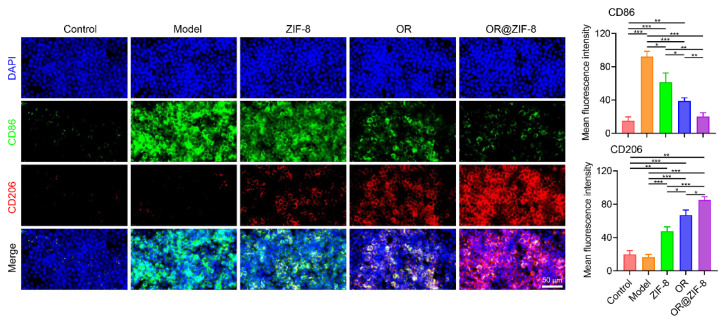
In vitro expressions of CD86 (green) and CD206 (red) (scale = 50 μm, n = 3, means ± SD), * *p* < 0.05, ** *p* < 0.01, and *** *p* < 0.001.

We verified the potential of the formulation to polarize macrophages towards the pro-repair M2 phenotype and regulate the immune microenvironment. During tissue repair, the polarization of macrophages from the pro-inflammatory M1 phenotype to the anti-inflammatory and pro-repair M2 phenotype is a key process for inflammation resolution and tissue regeneration [27]. In the Control group, the expression of CD86 (green fluorescence) and CD206 (red fluorescence) was weak (Figure 6). In the Model group, the fluorescence intensity of CD86 increased significantly, while the fluorescence intensity of CD206 remained at a low level. This suggests that injury induced the polarization of macrophages toward the M1 phenotype, with the pro-inflammatory response dominating. After treatment with ZIF-8 or OR, the fluorescence intensity of CD86 gradually decreased and the fluorescence intensity of CD206 gradually increased. Among these groups, the fluorescence intensity of CD86 in the OR@ZIF-8 group was significantly weaker than that in the Model, ZIF-8, and OR groups. In contrast, the fluorescence intensity of CD206 in the OR@ZIF-8 group was significantly stronger than that in the other groups. OR@ZIF-8 could effectively inhibit macrophage polarization toward the M1 phenotype and promote polarization toward the M2 phenotype. This creates an anti-inflammatory and pro-repair immune microenvironment for frostbite repair by regulating macrophage phenotype.

### 3.9. Physicochemical Characterization of OR@ZIF-8@HA Hydrogel

**Figure 7:**
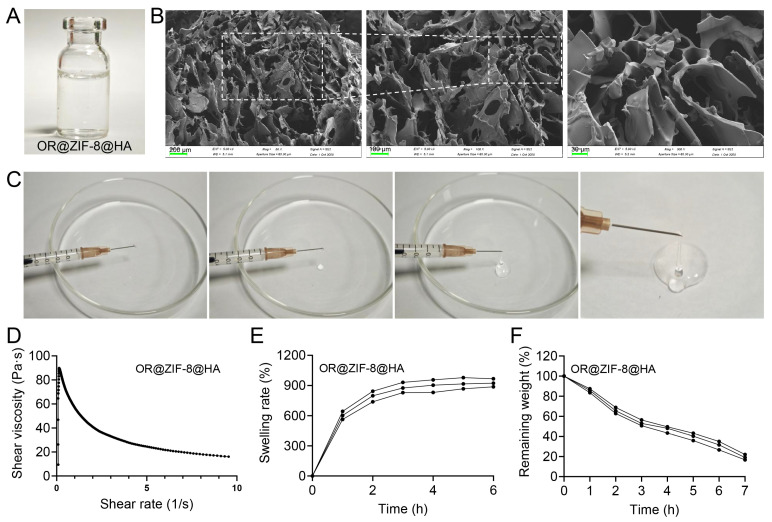
Morphological characteristics, injectability, and physicochemical property characterization of the OR@ZIF-8@HA. (**A**) Photographic appearance of the OR@ZIF-8@HA. (**B**) SEM microtopography of the OR@ZIF-8@HA. The dashed white line boxes in the left and middle subfigures denote the regions that are progressively magnified in the adjacent subsequent (middle and right) subfigures, respectively. Scale bars: 200 μm (left), 100 μm (middle), 30 μm (right). (**C**) Injectability characterization of the OR@ZIF-8@HA. (**D**) Rheological property of the OR@ZIF-8@HA. (**E**) Swelling rate over time curve of the OR@ZIF-8@HA. (**F**) In vitro degradation behavior of the OR@ZIF-8@HA.

To address the challenges of frequent administration and suboptimal bioavailability, we engineered the OR@ZIF-8@HA hydrogel by incorporating HA. Macroscopically, the OR@ZIF-8@HA hydrogel was homogeneous and transparent gel (Figure 7A), and devoid of visible stratification or particle sedimentation, underscoring the excellent dispersion of OR@ZIF-8 within the HA matrix. Scanning electron microscopy (SEM) of the lyophilized hydrogel revealed a canonical three-dimensional porous network structure (Figure 7B). Interconnected pores and corrugated lamellar pore walls were evident, and at higher magnifications, a continuous skeletal framework with open channels persisted. This morphology confirms the formation of a stable microporous architecture that is conducive to the exchange of wound exudates and localized drug delivery. Furthermore, injectability studies demonstrated facile extrusion through a needle (Figure 7C), indicating favorable injectability and moldability. Rheological characterization revealed a significant shear-thinning behavior for OR@ZIF-8@HA, with apparent viscosity decreasing markedly upon increasing shear rate (Figure 7D). This property suggests enhanced fluidity under shear stress, such as during injection or topical application, while a relatively high viscosity is maintained at rest, promoting sustained local retention. In terms of swelling behavior, the hydrogel exhibited rapid water uptake in aqueous environments (Figure 7E). The swelling ratio initially rapidly increased in the initial phase and subsequently plateaued within 3–6 h, There was minimal variation across replicates, attesting to the reproducibility of the swelling process. Concurrently, degradation experiments showed a progressive decrease in hydrogel mass over time (gradually decreasing from 0 to 7 h) (Figure 7F). This time-dependent erosion of the HA matrix in aqueous media provides a foundational basis for subsequent controlled release profiles and in situ cleanup within the wound environment.

### 3.10. In Vivo Observation of Frostbite Wound Healing Phenotype

**Figure 8:**
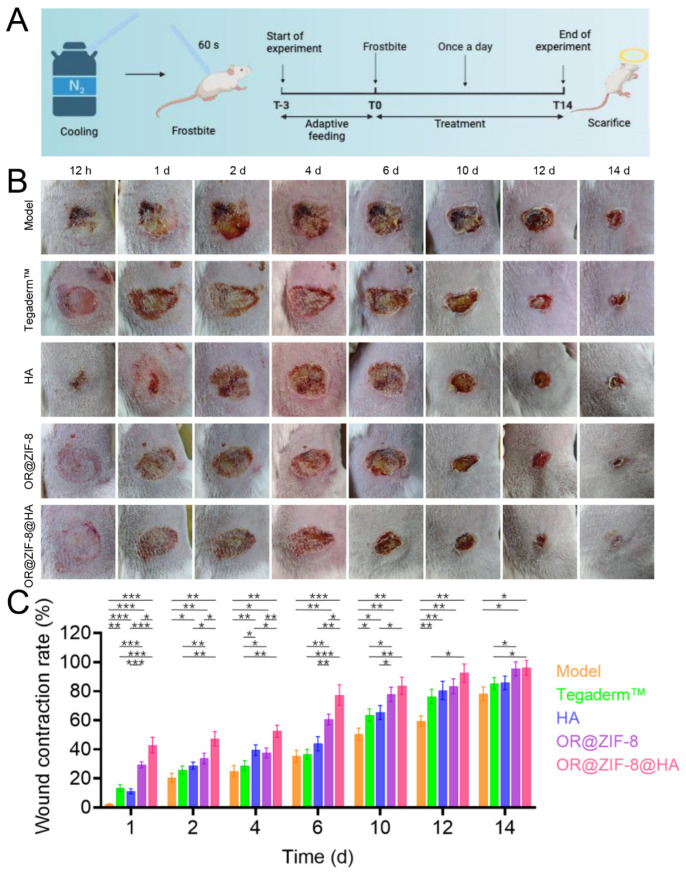
In vivo evaluation of OR@ZIF-8@HA in the frostbite mouse wound. (**A**) Establishment of the contact frostbite model and treatment process. (**B**) The Images of Different Treatments in Frostbitten Wounds from 12 h to 14 d. (**C**) Quantitative analysis of the wound contraction rate corresponding to the wounds shown in (**B**) (n = 6, means ± SD), * *p* < 0.05, ** *p* < 0.01, and *** *p* < 0.001.

We visually evaluated the overall therapeutic efficacy and wound healing outcomes of the formulation in an animal model. Although true frostbite occurs during slow tissue freezing and is difficult to simulate experimentally, in this study, we used a reproducible and easy-to-perform contact frostbite model [28] (Figure 8A). Using the mouse liquid nitrogen frostbite model, we visually observed the wound healing process of different treatment groups from 12 h to 14 d to evaluate the overall promotional effect of OR@ZIF-8@HA on frostbite repair. In the Model group, the wound showed obvious redness and scab formation starting from 12 h. A large area of the wound remained unhealed even at 14 d, indicating a slow repair process (Figure 8B). The wound healing rate of the Tegaderm^TM^ group and HA group was slightly faster than that of the Model group, but significant residual wounds were still visible at 12 d. The OR@ZIF-8 group showed improved rates of wound contraction and scab shedding. In the OR@ZIF-8@HA group, the degree of wound redness was weaker than that in the other treatment groups at 1 d. After 4 d, the scab area had decreased significantly. The wounds were largely closed at 10 d, while at 14 d, either only a very small residual wound remained or the wound was nearly completely healed. The healing rate in the OR@ZIF-8@HA group was 96.14 ± 4.12%, while that in the Tegaderm^TM^ group was 85.20 ± 4.15%. The OR@ZIF-8@HA formulation significantly accelerated the healing of frostbite wounds in mice. Its repair efficiency was superior to that of Tegaderm^TM^, which reflects the synergistic repair advantage of OR encapsulated in ZIF-8 and the HA matrix.

### 3.11. Histopathological Analysis

**Figure 9:**
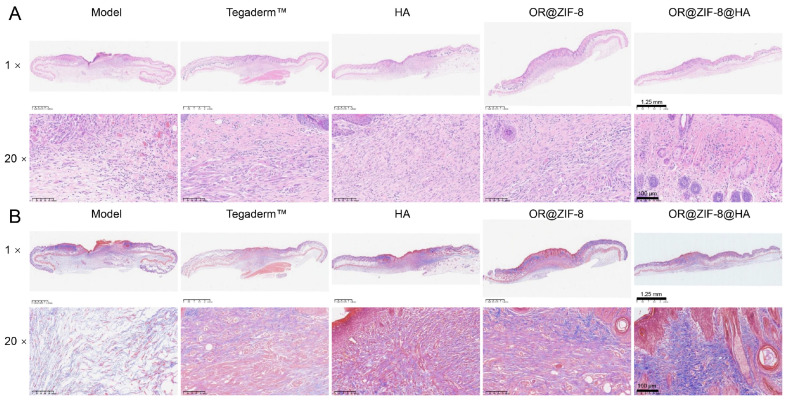
Histopathological analysis. (**A**) H&E staining images at 1× and 20× magnification, scale = 1.25 mm or 100 μm. (**B**) Masson staining images at 1× and 20× magnification, scale = 1.25 mm or 100 μm.

We microscopically assessed the quality of tissue repair and regeneration at the wound site, providing robust evidence for the therapeutic effect. Histological repair of frostbite wounds was evaluated via H&E and Masson staining. The H&E staining results are shown in Figure 9A. In the Model group, the wound exhibited obvious epidermal defects, severe dermal edema, massive inflammatory cell infiltration, and disorganized tissue morphology. The Tegaderm^TM^ and HA groups showed limited improvement in epidermal repair and inflammatory infiltration, with disordered fibrous hyperplasia remaining in the dermal layer. The OR@ZIF-8 group had accelerated epidermal regeneration and reduced inflammatory cell infiltration, but the regularity of the dermal collagen structure was insufficient. In contrast, the OR@ZIF-8@HA group had a nearly intact epidermis, significantly reduced inflammatory cell infiltration in the dermal layer, and a tissue arrangement more similar to normal skin. This indicates that OR@ZIF-8@HA has superior effects in terms of epidermal regeneration and inflammation regulation. The Masson staining results further showed (Figure 9B) that the collagen fibers in the Model group were sparse, fragmented, and disorganized. The Tegaderm^TM^ and HA groups had increased collagen fiber content, but the distribution remained less ordered. The OR@ZIF-8 group had increased collagen fiber density, but the arrangement regularity remained insufficient. The OR@ZIF-8@HA group had dense and neatly arranged collagen fibers that were more similar to the collagen structure of normal skin. This confirms that OR@ZIF-8@HA can more efficiently promote dermal collagen remodeling, providing a more solid histological support for wound healing.

**Figure 10:**
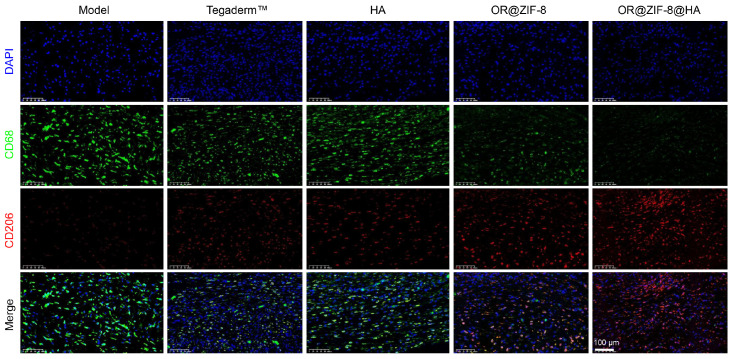
The immunofluorescence staining of CD206 (red), CD68 (green), and DAPI (blue), scale = 100 μm.

To further explore the mechanism by which the formulation regulates the wound microenvironment, the macrophage phenotype in the wound was detected. In the Model group, the wound showed high fluorescence intensity of CD68 and extremely weak fluorescence intensity of CD206, suggesting massive macrophage infiltration with a predominance of the M1 phenotype (Figure 10). The Tegaderm^TM^ group and HA group had slightly decreased CD68 fluorescence and slightly increased CD206 fluorescence, but the improvement was limited. The OR@ZIF-8 group had further reduced CD68 fluorescence and significantly increased CD206 fluorescence, with an elevated proportion of M2 phenotype. The OR@ZIF-8@HA group had the weakest CD68 fluorescence and the strongest CD206 fluorescence, with a significantly increased proportion of M2 phenotype. These results indicate that OR@ZIF-8@HA can effectively regulate macrophage polarization in frostbite wounds, promote their conversion to the anti-inflammatory and pro-repair M2 phenotype, and create a favorable immune microenvironment for wound healing.

## 4. Discussion

Current frostbite treatments, which rely on topical growth factors and conventional moist dressings, exhibit significant limitations [29]. Conventional growth factors suffer from short half-lives and dilution by wound exudate, hindering sustained therapeutic concentrations. Standard dressings, while providing initial moisture, lack active targeting to address the complex pathology of frostbite [29]. The cryoinjury-induced burst of ROS is central to the pathogenesis of frostbite; this triggers persistent inflammation that impedes epidermal regeneration and tissue repair [4]. Compounding this, persistent acidic microenvironments, which have been sustained for days by metabolites like lactate and CO_2_ from hypoxic conditions and inflammatory cell activity, pose a critical challenge [4,25].

Addressing this pH-dependent pathology, our study engineered an oxidation-resistant resveratrol OR@ZIF-8 nanocarrier. Leveraging the imidazole framework of ZIF-8 for pH-sensitive protonation and degradation in acidic conditions, we achieved sustained OR release precisely matched to the frostbite wound’s microenvironment, enabling pH-responsive drug delivery [30]. In vitro drug release studies under simulated frostbite conditions (pH 5.5) demonstrated a remarkable 75.46 ± 3.68% OR release within 36 h, confirming high pH responsiveness. Furthermore, ZIF-8’s high encapsulation efficiency mitigated OR’s poor water solubility and susceptibility to oxidation, ensuring robust bioactivity.

To further enhance therapeutic efficacy and user experience, OR@ZIF-8 nanoparticles were encapsulated within a HA hydrogel, forming the OR@ZIF-8@HA composite system. This integrated system benefits from HA hydrogel’s dual advantages: superior exudate absorption prolongs nanoparticle retention on the wound, preventing rapid dilution [31,32], and its inherent moistening properties counteract the desiccation issues seen with other Metal–Organic Framework-based carriers [33,34,35]. Crucially, it overcomes the deficit of active targeting drug release inherent in plain HA hydrogels [31,32]. This composite formulation’s superior performance was corroborated using in vivo studies, which demonstrated prolonged retention and significantly enhanced healing compared to controls and Tegaderm^TM^, validating its sustained efficacy in the acidic frostbite milieu.

The OR@ZIF-8@HA system exhibits multifaceted synergistic mechanisms, surpassing isolated antioxidant or anti-inflammatory strategies. Its efficacy stems from the polyphenol structure of OR undergoing redox reactions with ROS, which mitigates oxidative damage [36]. Simultaneously, the zinc ions released from ZIF-8 degradation synergistically promote wound healing, establishing a unique therapeutic modality beyond traditional gels [37]. ZIF-8’s advantage lies in its exceptional drug loading capacity, achieved via coordination, hydrogen bonding, and physical adsorption, facilitating the construction of multimodal synergistic systems [38]. In vitro, OR@ZIF-8 demonstrated superior scavenging of DPPH, ABTS^+^, and ·OH radicals compared to free OR. In an H_2_O_2_-induced HaCaT cell oxidative injury model, it preserved cell viability and promoted migration. It also modulated inflammatory cytokine levels (reduced TNF-α, IL-6; elevated IL-10 and polarized macrophages towards an M2 pro-repair phenotype. Crucially, zinc ions, which are essential cofactors for enzymes like prolyl hydroxylase, lysyl hydroxylase, and lysyl oxidase, ensure collagen synthesis and cross-linking, underpinning structural integrity and promoting re-epithelialization via MAPK/ERK pathway activation [39]. Furthermore, zinc ions exhibit broad-spectrum antibacterial activity by disrupting bacterial cell membranes and physiological processes [39], complementing OR’s adjuvant antimicrobial effects [40]. The HA hydrogel matrix provides a physical barrier that protects against infection.

Despite these advances, certain limitations warrant further investigation. The broad-spectrum antimicrobial activity of OR@ZIF-8@HA against common frostbite pathogens requires systematic evaluation. While HA hydrogel offers good biocompatibility and moisture retention, its parameters (molecular weight, crosslinking density) need to be optimized for frostbite wounds [32]. Systematic in vitro compatibility and functional verification will be conducted in other cell types, such as fibroblasts, to comprehensively assess the system’s impact on different cell populations throughout the wound healing process. Recognizing that the OR@ZIF-8@HA hydrogel is designed for extemporaneous clinical use, prepared freshly prior to application rather than as a long-term stored commercial formulation, future research could explore alternative delivery systems like microneedles [41], validate safety and efficacy in large animal models, and investigate the utility of this type of treatment for other chronic wound types.

## 5. Conclusions

In this study, the developed nanocomposite hydrogel achieved efficient delivery of OR through the pH-responsive release of ZIF-8 and the long-term retention and moisturizing properties of HA. Meanwhile, via a multi-target mechanism that includes protecting keratinocytes, regulating inflammation and macrophage polarization, and promoting cell migration and collagen remodeling, the composition significantly improved the healing process of frostbite wounds. This study provides a novel multifunctional composite formulation for frostbite treatment. Additionally, it offers theoretical and experimental bases for the design of topical drug delivery systems based on nanocomposite hydrogels. Furthermore, it lends support to wound repair strategies involving synergistic regulation of multiple pathological links.

## Data Availability

The data that support the findings of this study are available in the manuscript. Extra data used to support this study are available from the corresponding author upon request.

## Abbreviations

The following abbreviations are used in this manuscript:

OH	Hydroxyl Radical
ABTS+	2,2′-Azino-bis(3-ethylbenzthiazoline-6-sulfonic acid)
CCK-8	Cell Counting Kit-8
CD206	Cluster of Differentiation 206
CD86	Cluster of Differentiation 86
DL	Drug Loading
DLS	Dynamic Light Scattering
DPPH	1,1-Diphenyl-2-picrylhydrazyl
EE	Encapsulation Efficiency
H2O2	Hydrogen Peroxide
HA	Hyaluronic Acid
HaCaT	Human Immortalized Keratinocyte Cell Line
IL-10	Interleukin-10
IL-6	Interleukin-6
LPS	Lipopolysaccharide
OR	Oxyresveratrol
OR@ZIF-8	Oxyresveratrol@Zeolitic Imidazolate Framework-8
RAW264.7	Macrophage Cell Line
TEM	Transmission Electron Microscope
TNF-α	Tumor Necrosis Factor-α

## References

[1] Sheridan R.L., Goverman J.M., Walker T.G. (2022). Diagnosis and Treatment of Frostbite. N. Engl. J. Med..

[2] Regli I.B., Strapazzon G., Falla M., Oberhammer R., Brugger H. (2021). Long-Term Sequelae of Frostbite—A Scoping Review. Int. J. Environ. Res. Public Health.

[3] Magnan M.-A., Gayet-Ageron A., Louge P., Champly F., Joffre T., Lovis C., Pignel R. (2021). Hyperbaric Oxygen Therapy with Iloprost Improves Digit Salvage in Severe Frostbite Compared to Iloprost Alone. Mex.

[4] Li X., Pan J., Liu M., Zhang E., Li Y., He Y., Yang G., Zhou S. (2025). Design and Development of Fiber Patch to Prevent Reperfusion Injury and Provide Thermal Stimulation for Treating Severe Frostbite. Adv. Funct. Mater..

[5] Joshi K., Goyary D., Mazumder B., Chattopadhyay P., Chakraborty R., Bhutia Y.D., Karmakar S., Dwivedi S.K. (2020). Frostbite: Current Status and Advancements in Therapeutics. J. Therm. Biol.

[6] Turner B.L., Dongen T.T.C.F.v., Berendsen R.R., Jong F.J.M.d., Endert E.L., van Hulst R.A., Hoencamp R. (2025). Frostbite: A Treatment Guideline for Prehospital Treatment in a Military Environment. BMJ Mil. Health.

[7] Li T., Gao D., Wang W., Li X., Han M., Ma J., Zhu Y. (2025). Establishment and Identification of a Deep Second-Degree Frostbite Model in Mouse Skin. Ther. Hypothermia Temp. Manag..

[8] Wang W., Liu P., Zhu W., Li T., Wang Y., Wang Y., Li J., Ma J., Leng L. (2025). Skin Organoid Transplantation Promotes Tissue Repair with Scarless in Frostbite. Protein Cell.

[9] Li S., Li W., Wu X., Zhang B., Liu L., Yin L. (2024). Immune Cell-Derived Extracellular Vesicles for Precision Therapy of Inflammatory-Related Diseases. J. Control. Release.

[10] Priyono Q.A.P., Yusniasari P.A., Alifiansyah M.R.T., Suryanto G.Y., Widyowati R., Herdiansyah M.A., Ansori A.N.M., Purnobasuki H., Pratiwi I.A., Makhmudov F. (2024). Ethnomedical Potentials, Phytochemicals, and Medicinal Profile of Alpinia Galanga L.: A Comprehensive Review. BIO Integr..

[11] Shen C., Xu P., Shen B., Min H., Li X., Han J., Yuan H. (2016). Nanogel for Dermal Application of the Triterpenoids Isolated from Ganoderma Lucidum (GLT) for Frostbite Treatment. Drug Deliv..

[12] Passos C.L.A., Ferreira C., De Carvalho A.G.A., Silva J.L., Garrett R., Fialho E. (2024). Oxyresveratrol in Breast Cancer Cells: Synergistic Effect with Chemotherapeutics Doxorubicin or Melphalan on Proliferation, Cell Cycle Arrest, and Cell Death. Pharmaceutics.

[13] Shi X., Xu L., Zhang J., Mo J., Zhuang P., Zheng L. (2023). Oxyresveratrol from Mulberry Branch Extract Protects HUVECs against Oxidized Low-Density Lipoprotein-Induced Oxidative Injury via Activation of the Nrf-2/HO-1 Pathway. J. Funct. Foods.

[14] Suriyaprom S., Srisai P., Intachaisri V., Kaewkod T., Pekkoh J., Desvaux M., Tragoolpua Y. (2023). Antioxidant and Anti-Inflammatory Activity on LPS-Stimulated RAW 264.7 Macrophage Cells of White Mulberry (*Morus alba* L.) Leaf Extracts. Molecules.

[15] Yu H.-J., Liu J.-H., Liu W., Niu R., Zhang B., Xiong Y., Liu Y., Wang Y.-H., Zhang H.-J. (2025). Metal-Based Nanomedicines for Cancer Theranostics. Mil. Med. Res..

[16] de Alencar Filho J.M.T., França A.R.d.S., da Silva L.B.R., Sampaio P.A., Pereira E.C.V., da Silva A.R., Alencar M.V.V.d.O., Araújo T.C.d.L., Menezes P.M.N., Costa S.P.M. (2025). Vitexin as a Potential Antidysmenorrheic Agent: Development of a ZIF-8-Based Immediate-Release System and Evaluation via In Vivo and In Silico Approaches. Biomedicines.

[17] Wang S., Liu Y., Wang X., Chen L., Huang W., Xiong T., Wang N., Guo J., Gao Z., Jin M. (2024). Modulating Macrophage Phenotype for Accelerated Wound Healing with Chlorogenic Acid-Loaded Nanocomposite Hydrogel. J. Control. Release.

[18] Piao Y., Wang N., Jin M., Piao J., Han M., Wang Z., Quan C., Yin J., Gao Z., Cui W. (2025). Multi-Trace Elements-Enriched Functional Drink Accelerates Gastric Ulcer Repair via the HGF/c-Met/STAT3 Pathway. J. Funct. Foods.

[19] Chen Y., Wang X., Tao S., Wang Q., Ma P.-Q., Li Z.-B., Wu Y.-L., Li D.-W. (2023). Research Advances in Smart Responsive-Hydrogel Dressings with Potential Clinical Diabetic Wound Healing Properties. Mil. Med. Res..

[20] Wójcik-Pastuszka D., Iwaszkiewicz R., Musiał W. (2024). The Effects of Synthetic Polymers on the Release Patterns of Bupivacaine Hydrochloride from Sodium Hyaluronate Hydrogels. Biomedicines.

[21] Wang S.-Q., Jin M.-J., Guo Z.-K., Shen D.-R., Jin L.-N., Cheng F., Zhao Y.-R., Liu T., Li Y.-C., Wang N.-Y. (2025). Trace Element-Dictated Exosome Modules and Self-Adaptive Dual-Network Hydrogel Orchestrate Diabetic Foot Regeneration through Complement-Mitochondria-Autophagy Circuitry. Mil. Med. Res..

[22] Nishad M., Verma S., Kumar A., Khurana N. (2025). Formulation and Evaluation of Apigenin-Loaded Microsponge Gel for Effective Angioedema Therapy. Curr. Pharm. Anal..

[23] Wan H., Wang S., Li C., Zeng B., Wu H., Liu C., Chen L., Jin M., Huang W., Zang Y. (2023). LA67 Liposome-Loaded Thermo-Sensitive Hydrogel with Active Targeting for Efficient Treatment of Keloid via Peritumoral Injection. Pharmaceutics.

[24] Zhang Z., Ma S.-Y., Yin X., Li Y.-S., Tang H.-B. (2025). Topical Frankincense Treatment for Frostbite Based on Microcirculation Improvements. J. Ethnopharmacol..

[25] Yi M., Jin W., Li H., Niu X., Wang J., Wang S., Zhang W., Zhou M., Wang Z., Zhou Y. (2025). pH-Responsive Bilayer Hydrogel with Sequential Release of Morin-Based Nanoparticles and bFGF for the Treatment of the “Ice and Fire” Wounds. Mater. Today Bio.

[26] The ARRIVE Guidelines 2.0 | ARRIVE Guidelines. https://arriveguidelines.org/arrive-guidelines.

[27] Zhou J., Jiang S., Wang L., Lin K., Wu J., Gui H., Gao Z. (2025). Decellularized Adipose Matrix Rejuvenates Photoaged Skin through Immune Microenvironment Modulation. BME Front..

[28] Volkova M.V., Boyarintsev V.V., Trofimenko A.V., Kovaleva E.V., Othman A.A., Melerzanov A.V., Filkov G.I., Rybalkin S.P., Durymanov M.O. (2023). Local Injection of Bone-Marrow Derived Mesenchymal Stromal Cells Alters a Molecular Expression Profile of a Contact Frostbite Injury Wound and Improves Healing in a Rat Model. Burns.

[29] Wibbenmeyer L., Lacey A.M., Endorf F.W., Logsetty S., Wagner A.L.L., Gibson A.L.F., Nygaard R.M. (2024). American Burn Association Clinical Practice Guidelines on the Treatment of Severe Frostbite. J. Burn Care Res..

[30] Sun Y., Zheng L., Yang Y., Qian X., Fu T., Li X., Yang Z., Yan H., Cui C., Tan W. (2020). Metal–Organic Framework Nanocarriers for Drug Delivery in Biomedical Applications. Nano-Micro Lett..

[31] Jia S., Zhou H., Chen J., Lin J., Zhu X., Weng J., Li W., Yu F. (2025). A Comprehensive Review of Hydrogel Strategies for Repairing Peripheral Nerve Injuries. Brain-X.

[32] Wu L., Zhou X., Cheng W., Pan X., Wang C.H., Li Z.Y., Yang Y.H., Zong D.S. (2025). Current Applications and Future Developments in Biomedical Hydrogels. J. Shenyang Pharm. Univ..

[33] Wu X., Meng F., Lin Z., Sun Y., Li H., Zhang S., Bian B., Su W., Wang X., Liu H. (2025). A Tissue Regeneration-Promoting Hydrogel Dressing Incorporating Zirconium MOF Loaded with Curcumin for Multi-Modal Healing of Bacterial-Infected Wounds. Mater. Today Chem..

[34] Singh H., Dan A., Prasanna Kumari B., Dave H., Parsaila N., Navale A., Darban Z., Yadav I., Goyal P., Misra S.K. (2024). Copper-MOF and Tannic Acid-Empowered Composite Cryogel as a Skin Substitute for Accelerated Deep Wound Healing. Biomater. Adv..

[35] Wu X., Liu Q., Rasiuk A., Vladimirovich S.S., Pan Y., Lin B., Liu Y., Li W., Li G., Li W. (2025). Polysaccharide-Based Aerogel Dressing Integrated with MOF-Loaded Quercetin with Multifunctional Effects for Promoting Infected Wound Healing. APL Mater..

[36] Fayazbakhsh F., Hataminia F., Eslam H.M., Ajoudanian M., Kharrazi S., Sharifi K., Ghanbari H. (2023). Evaluating the Antioxidant Potential of Resveratrol-Gold Nanoparticles in Preventing Oxidative Stress in Endothelium on a Chip. Sci. Rep..

[37] Banerjee D., Vishwakarma S., Nayak M., Upadhyay A., Pradhan L., Makam P., Mukherjee S. (2025). Fabrication of Zinc-Tryptophan Nanoassemblies for Antibacterial and Wound Healing Applications. ACS Appl. Nano Mater..

[38] Guo Y., Sun Q., Wu F.-G., Dai Y., Chen X. (2021). Polyphenol-Containing Nanoparticles: Synthesis, Properties, and Therapeutic Delivery. Adv. Mater..

[39] Zhao Y., Liu J., Sun L., Liu H., Chen X., Deng X., Ping Y., Han W., Wang J., Tian F. (2025). Zinc-Doped Curcumin Carbon Dots Promote Infected Wound Healing with Photodynamic via the VEGF Signaling Pathway. J. Nanobiotechnol..

[40] Rahbardar M.G., Kesharwani P., Sahebkar A. (2025). Resveratrol in Anti-Biofilm Therapy: Mechanisms, Molecular Derivatives, and Emerging Drug Delivery Strategies. Fitoterapia.

[41] Sabbagh F., Kim B.S. (2022). Microneedles for Transdermal Drug Delivery Using Clay-Based Composites. Expert Opin. Drug Deliv..

